# Impact of 3-Amino-1,2,4-Triazole (3-AT)-Derived Increase in Hydrogen Peroxide Levels on Inflammation and Metabolism in Human Differentiated Adipocytes

**DOI:** 10.1371/journal.pone.0152550

**Published:** 2016-03-29

**Authors:** Francisco Javier Ruiz-Ojeda, Carolina Gomez-Llorente, Concepción María Aguilera, Angel Gil, Azahara Iris Rupérez

**Affiliations:** 1 Department of Biochemistry and Molecular Biology II, Institute of Nutrition and Food Technology "José Mataix", Center of Biomedical Research, University of Granada, Avda. del Conocimiento s/n., Armilla, Granada, 18016, Spain; 2 Instituto de Investigación Biosanitaria IBS.GRANADA, Granada, Spain; 3 CIBER Fisiopatología de la Obesidad y la Nutrición (CIBEROBN), Madrid, Spain; Tohoku University, JAPAN

## Abstract

Obesity is characterized by an excessive accumulation of fat in adipose tissue, which is associated with oxidative stress and chronic inflammation. Excessive H_2_O_2_ levels are degraded by catalase (CAT), the activity of which is decreased in obesity. We investigated the effects of inhibition of catalase activity on metabolism and inflammation by incubating human differentiated adipocytes with 10 mM 3-amino-1,2,4-triazole (3-AT) for 24 h. As expected, the treatment decreased CAT activity and increased intracellular H_2_O_2_ levels significantly. Glutathione peroxidase (GPX) activity was also reduced, and the gene expression levels of the antioxidant enzymes *GPX4* and peroxiredoxins (1, 3 and 5) were inhibited. Interestingly, this occurred along with lower mRNA levels of the transcription factors nuclear factor (erythroid 2-like 2) and forkhead box O, which are involved in redox homeostasis. However, superoxide dismutase activity and expression were increased. Moreover, 3-AT led to nuclear factor kappa-light-chain-enhancer of activated B cells (NF-κB) activation and increased tumor necrosis alpha and interleukin 6 protein and gene expression levels, while lowering peroxisome proliferator-activated receptor gamma (*PPAR*γ) mRNA and protein levels. These alterations were accompanied by an altered glucose and lipid metabolism. Indeed, adipocytes treated with 3-AT showed reduced basal glucose uptake, reduced glucose transporter type 4 gene and protein expression, reduced lipolysis, reduced AMP-activated protein kinase activation and reduced gene expression of lipases. Our results indicate that increased H_2_O_2_ levels caused by 3-AT treatment impair the antioxidant defense system, lower *PPARγ* expression and initiate inflammation, thus affecting glucose and lipid metabolism in human differentiated adipocytes.

## Introduction

Obesity is a global concern for societies and healthcare systems [1], and its prevalence worldwide needs to be decreased. In obese adipose tissue, the adipokine secretion profile is altered, there is low-grade inflammation and lipid and glucose metabolism are affected [2,3]. In addition, obese adipose tissue has also been characterized by an excessive production of reactive oxygen species (ROS) [4].

An imbalance between ROS production and scavenging mechanisms leads to the development of oxidative stress in adipose tissue, which is associated with the previously mentioned metabolic alterations [4,5,6]. In particular, one of the most abundant forms of ROS in adipocytes is hydrogen peroxide (H_2_O_2_), the levels of which are heavily regulated by different enzymes that include catalase (CAT), glutathione peroxidases (GPX), superoxide dismutase (SOD) and peroxiredoxins (PRDXs) [7]. Although H_2_O_2_ is an important signaling molecule at controlled levels [8,9], its increased production can determine metabolic alterations in adipocytes [10].

Interestingly, catalase activity, which is responsible for the degradation of excessive amounts of H_2_O_2_, has been shown to be decreased in obese adults [11] as well as in children with obesity and insulin resistance [12,13]. Moreover, obese and type 2 diabetic mice have lower *CAT* expression and higher H_2_O_2_ levels in adipose tissue [4,14].

It is known that oxidative stress can activate the nuclear factor kappa-light-chain-enhancer of activated B cells (NF-κB) inflammation pathway. Serine phosphorylation at various sites of the NF-κB p65 subunit has been shown to be important for the transcription of various inflammatory mediators, including tumor necrosis factor alpha (TNF-α) and interleukin 6 (IL-6) [15,16]. TNF-α is a potent cytokine with many adverse effects such as insulin resistance [17] and activation of lipolysis [18].

Additionally, ROS are also able to lower peroxisome proliferator-activated receptor gamma (*PPARγ*) expression [19], which can itself regulate *CAT* expression in adipose tissue [20]. In fact, in obese individuals, treatment with rosiglitazone, a PPARγ agonist, increased CAT protein levels in adipose tissues [21]. Along with this finding, it has been observed that oxidative stress can lead to a down-regulation of adiponectin (*ADIPOQ*) and glucose transporter 4 (*GLUT4*) gene expression in adipose tissue [4,6].

Regarding the effects of oxidative stress on lipid metabolism, H_2_O_2_ has been shown to inhibit cAMP-stimulated protein kinase A (PKA) activity, thus reducing lipolysis in adipocytes [14]. Regarding 5'-AMP-activated protein kinase (AMPK), it has been shown to be activated as a consequence of lipolysis, in parallel with an increase in oxidative stress [22]. Furthermore, oxidative stress can inhibit the expression of lipase genes by activating inflammation and reducing *PPARγ* expression.

Regarding the cellular responses to oxidative stress, nuclear factor (erythroid 2-like 2) (Nrf2) and forkhead box O (FOXO1) play important roles in maintaining intracellular redox homeostasis by inducing the expression of antioxidant enzymes [23,24]. Moreover, it has been reported that ROS can modulate the Wnt/β-catenin pathway and that low levels of oxidative stress favor the interaction of β-catenin with FOXO1 to protect the cell against oxidative damage [25].

Although these facts make it clear that oxidative stress is associated with inflammation, insulin resistance and altered lipid metabolism, the exact contribution of catalase activity to the protection against the progression of these metabolic alterations is not clear. Thus, by using the irreversible CAT inhibitor 3-amino-1,2,4-triazole (3-AT) [26] in human differentiated adipocytes, we investigated the mechanism by which catalase activity contributes to the deleterious effects of oxidative stress in adipose tissue.

## Materials and Methods

### Materials

Adipose derived-stem cells (ADSCs) were purchased directly from Lonza (Poietics™ Normal ADSCs, Lonza, PT-5006, Lot 0F4505, Switzerland). These commercially available ADSCs are isolated from normal (non-diabetic) adult subcutaneous lipoaspirates collected during elective surgical liposuction procedures. ADSCs have been reported to differentiate into many different lineages, including chondrogenic, osteogenic, adipogenic and neural lineages. Adipogenesis media and reagents were obtained from Lonza, and 3-AT was purchased from Sigma (Sigma-Aldrich, St. Louis, MO, USA). Adenosine 3′,5′-cyclic monophosphate (cAMP) was acquired from Sigma (A9501). The rabbit anti-GLUT4 antibody (H-61) and TNF-α antibody (SC-52746) were acquired from Santa Cruz Biotechnology (Santa Cruz, CA, USA). The goat anti-adiponectin antibody (AF1065) was obtained from R&D Systems (R&D, Inc. USA). The rabbit anti-PPARγ (D69), phosphor-NF-kB p65 (Ser536), rabbit anti-total AMPKα (D5A2) and rabbit anti-phospho-AMPKα (Thr172) antibodies were acquired from Cell Signaling Technologies (Beverly, MA, USA). The mouse anti-α-tubulin antibody (T5158) and horseradish peroxidase-conjugated immunoglobulin were purchased from Sigma. Unless otherwise indicated, all other chemicals were purchased from Sigma.

### Cell culture and incubation

The ADSCs were cultured, expanded and differentiated into adipocytes according to the manufacturer’s recommendations. Briefly, ADSCs were grown and expanded in appropriate sterile plastic dishes in complete Advanced-DMEM medium (Gibco, Life Technologies, Carlsbad, CA, USA) supplemented with 2 mM L-glutamine (25030, Gibco, Life Technologies, Carlsbad, CA, USA), 10% fetal bovine serum (FBS, PT-9000 H, Lonza, Basel, Switzerland), 100 U ml^-1^ penicillin and 100 μg ml^-1^ streptomycin (10378–016, Gibco, Life Technologies, Carlsbad, CA, USA). Cells were incubated at 37°C in a humidified atmosphere containing 5% CO_2_. The cell culture medium was replaced twice per week, and cells were passaged up to a maximum of 6 times. To induce differentiation, cells were seeded in 35-mm dishes at a density of 30,000 cells/cm^2^ and cultured in preadipocyte growth medium (PGM) consisting of Preadipocyte Basal Medium-2 (PT-8002, Lonza) supplemented with 10% FBS, 2 mM L-Glutamine (PT-9001 H, Lonza) and 0.1 μg/ml gentamicin sulfate/amphotericin-B (PT-4504, Lonza). At 90% confluency, the growth medium was replaced with differentiation medium (PGM supplemented with dexamethasone, 3-isobutyl-1-methylxanthine, indomethacin and h-insulin; PT-9502, Lonza). ADSCs were incubated with differentiation medium for 10 days. Finally, cells were washed twice with PBS, and the differentiation medium was replaced with PGM overnight. Adipogenesis was monitored and quantified by morphological examination of the cellular accumulation of lipid droplets by Oil Red O staining (234117, Sigma-Aldrich, St. Louis, MO, USA; Fig A in S1 File) and by spectrophotometric determination of washed Oil Red O staining (Fig B in S1 File). All treatments were performed on differentiated adipocytes on day 10.

### Catalase activity assay

Human differentiated adipocytes were incubated in the presence or absence of 3-AT (2 mM and 10 mM) for 24 h, and the catalase activity was determined in cell lysates using a colorimetric assay (K033-H1, Arbor assays, Michigan, USA). Briefly, the cells were harvested, lysed with protein lysis buffer (PLB) containing 10 mM Tris-HCl (pH 7.5), 150 mM NaCl, 2 mM EDTA, 1% Triton X-100, 10% glycerol and protease inhibitor cocktail (Thermo Scientific, Massachusetts, USA), and placed on ice for 20 min. Then, the cell lysates were centrifuged (30 min, 13000 ×*g*, 4°C), and the supernatants were used to determine the protein concentrations with the Protein Assay Kit II (Bio-Rad Laboratories, California, USA), which was performed according to the manufacturer’s instructions. A bovine catalase standard was used to generate a standard curve for the assay. Next, hydrogen peroxide was added to the supernatants, and they were incubated at room temperature for 30 min. The HRP reacts with the substrate in the presence of hydrogen peroxide to convert the colorless substrate into a pink-colored product. All samples were compared to the standard curve, and the activity of catalase in each sample was calculated after making the appropriate corrections for dilutions, using the software available with the plate reader. The results were presented as units of catalase activity per mg protein. Sensitivity was determined to be 0.052 U/mL, and the limit of detection was determined to be 0.062 U/mL.

### Intracellular H_2_O_2_ determination

The generation of intracellular H_2_O_2_ in the presence or absence of 3-AT (2 mM and 10 mM) for 24 h by adipocytes was measured in cell lysates using the OxiSelect fluorometric assay (Cell Biolabs, San Diego, CA, USA). Cell lysates were incubated, and the fluorescence was measured with a microplate reader in standard 96-well fluorescence black microtiter plates using an excitation wavelength of 530 nm and a detection wavelength of 590 nm. Intracellular H_2_O_2_ results were expressed as μM of H_2_O_2_.

### Superoxide dismutase and glutathione peroxidase activity assays

SOD and GPX activities were determined spectrophotometrically in cell lysates of adipocytes in the presence or absence of 3-AT (10 mM) for 24 h using two commercial kits (K028-H1 for SOD, Arbor assays, Michigan, USA; 703102 for GPX, Cayman Chemical, Michigan, USA). Samples were harvested with PLB, diluted in the buffer diluents and then added to the wells with the rest of the reagents. The SOD activity assay was performed according to the manufacturer’s instructions. The absorbance was measured at 450 nm, and the results were expressed in terms of the units of SOD activity per mg protein. Sensitivity was determined to be 0.044 U/mL, and the limit of detection was determined to be 0.0625 U/mL. GPX activity was measured indirectly by a coupled reaction with glutathione reductase (GR). Oxidized glutathione (GSSG), which is produced upon reduction of cumene hydroperoxide by GPX, is recycled to its reduced state by GR and NADPH. The oxidation of NADPH to NADP+ is accompanied by a decrease in absorbance at 340 nm. Thus, the rate of decrease is directly proportional to the GPX activity in the sample. The results were expressed in nmol/min/mg protein. Sensitivity was determined to be 0.02 of decreased absorbance per minute, and the limit of detection was determined to be 50 nmol/min/mL.

### GSH/GSSG ratio detection assay

Reduced and oxidized glutathione GSH/GSSG ratio of cell lysates was measured with a fluorometric kit (ab138881, Abcam, Cambridge, UK). GSH and total glutathione were determined by changes in fluorescence intensity, and GSSG concentration was calculated using total glutathione–GSH. The results were expressed as GSH/GSSG ratios in the presence or absence of 3-AT (10 mM) for 24 h in human differentiated adipocytes.

### Lipolysis assay

The total glycerol release, the final product of lipolysis, was measured in cell supernatants using a colorimetric assay (Free Glycerol Reagent, F6428, Sigma, St. Louis, MO, USA). The cells were treated with 3-AT (10 mM) for 24 h, and the cell supernatants were harvested. Then, the cell supernatants were incubated in the reagent at room temperature for 15 min in a 96-well plate, and the optical density at 550 nm was measured using a microplate reader (BioTek HTX, Fischer Scientific, USA).

### RNA isolation and qRT-PCR

Total RNA was extracted from cells using the PeqGOLD HP Total RNA kit (Peqlab, Germany). Isolated RNA was treated with Turbo DNase (Ambion, Life Technologies, Carlsbad, CA, USA). The final RNA concentration and quality were determined using a NanoDrop2000 (NanoDrop Technologies, Winooski, Vermont, USA). Total RNA (500 ng) was transcribed into cDNA using the iScript cDNA Synthesis Kit (Bio-Rad Laboratories, California, USA). Differential gene expression levels of *CAT*, hormone sensitive lipase (*HSL*), adipose triglyceride lipase (*ATGL*), *PPARγ*, adipocyte fatty acid-binding protein (*FABP4*), and perilipin (*PLIN*) were determined by qPCR using specific primer sequences. Glyceraldehyde 3-phosphate dehydrogenase (*GAPDH*) was used as a reference gene for the differentiation experiments, while hypoxanthine-guanine phosphoribosyltransferase-1 (*HPRT1*) was used for 3-AT treatment experiments as *GAPDH* is not a suitable reference gene when glucose metabolism is altered. The specific primer sequences were designed using Primer3 (http://bioinfo.ut.ee/primer3-0.4.0/, Table 1). Primers for *GLUT4*, *IL-6*, *TNF-α*, *NFKB2*, tumor necrosis factor receptor superfamily, member 1A (*TNFRSF1A*), glutathione peroxidase 4 (*GPX4*), peroxiredoxin 1 (*PRDX1*), peroxiredoxin 3 (*PRDX3*), peroxiredoxin 5 (*PRDX5*), catenin beta 1 (*CTNNB1*), *FOXO1*, *NRF2* and superoxide dismutase 1, soluble (*SOD1*) were obtained from Bio-Rad Laboratories, California, USA. The qPCR was performed with an ABI Prism 7900 instrument (Applied Biosystems, Foster City, CA, USA) using SYBR Green PCR Master Mix (Applied Biosystems, Foster City, CA, USA). Quantification was performed using the Pfaffl method [27]. Compliance with the minimum information for publication of quantitative real-time PCR experiments (MIQE) was made possible using Bio-Rad’s PrimePCR assays. Statistical validation of the stability of the reference genes was calculated in each sample. Bio-Rad recommends using a <0.5 value, which is the most stable expression in the tested samples. The results are expressed as fold-change calculated using the 2^^-ΔΔC^t method.

**Table 1:** Forward and reverse primer sequences used in the qPCR assays.

Gene	Primer sequence		
	Forward	Reverse	Size (bp)
*CAT*	5’-GCCTGGGACCCAATTATCTT-3’	5’-GAATCTCCGCACTTCTCCAG-3’	203
*HSL*	5’-CTTCTGGAAAGCCTTCTGGAACATCACCGA-3’	5’-CTGAGCTCCTCACTGTCCTGTCCTTCAC-3’	249
*ATGL*	5’-GACGAGCTCATCCAGGCCAATGTCTG-3’	5’-GATGGTGTTCTTAAGCTCATAGAGTGGCAGG-3’	141
*PPARγ*	5’-CTCGAGGACACCGGAGAGG-3’	5’-CACGGAGCTGATCCCAAAGT-3’	121
*FABP4*	5’-GCTTTTGTAGGTACCTGGAAACTT-3’	5’-ACACTGATGATCATGTTAGGTTTGG-3’	125
*PLIN*	5’-CTCTCGATACACCGTGCAGA-3’	5’-tggtcctcatgatcctcctc-3’	207
*GAPDH*	5’GAGTCAACGGATTTGGTCGT-3’	5’-TTGATTTTGGAGGGATCTCG-3’	238
*HPRT1*	5’-GAGATGGGAGGCCATCACATTGTAGCCCTC-3’	5’-CTCCACCAATTACTTTTATGTCCCCTGTTGACTGGTC-3’	76

CAT, Catalase; HSL, Hormone sensitive lipase; ATGL, Adipose triglyceride lipase; PPARγ, Peroxisome proliferator-activated receptor gamma; FABP4, Fatty acid binding protein 4, adipocyte; PLIN, Perilipin; GAPDH, Glyceraldehyde 3-phosphate dehydrogenase; HPRT1, Hypoxanthine-guanine phosphoribosyltransferase-1.

### Western blot assays

Protein samples from cell lysates containing 2.5 μg of protein were mixed with 3X SDS-PAGE sample buffer (100 mM Tris-HCl, pH 6.8, 25% SDS, 0.4% bromophenol blue, 10% β-mercaptoethanol and 2% glycerol), separated via SDS-PAGE using a TGX Any kD gel (Bio-Rad Laboratories, California, USA) and transferred to a nitrocellulose membrane (Bio-Rad Laboratories, California, USA). After incubation in blocking buffer [5% non-fat milk and 1% Tween 20 in Tris-buffered saline (TBS)], the membranes were probed with one of the following antibodies: anti-catalase (1:2000 in 5% non-fat milk), anti-GLUT4 (1:100 in 5% non-fat milk), anti-adiponectin (1:500 in 5% bovine serum albumin, BSA), anti-PPARγ (1:1000 in 5% BSA), anti-TNFα (1:100 in 5% BSA), anti-phospho-NF-kB p65 (Ser536) (1:500 in 5% BSA), anti-total AMPKα, anti-phosphorylated AMPKα (phospho-AMPKα T172) (both 1:1000 in 5% BSA) and anti-α-tubulin (internal control, 1:4000 in 5% non-fat milk). Immunoreactive signals were detected via enhanced chemiluminescence (Super-Signal West Dura Chemiluminescent Substrate, 34075, Thermo Scientific, Europe). The membrane images were digitally captured and the densitometric analyses were conducted using ImageJ software. The results were expressed as the fold-change in expression relative to the control.

### Intracellular IL-6 protein levels

The intracellular IL-6 levels were determined in cell lysates in the presence or absence of 10 mM 3-AT for 24 h. Samples were harvested with PLB, diluted in the buffer diluents and then added to the wells with the rest of the reagents. IL-6 was determined using a MILLI*plex*^TM^ kit (HADK2MAG-61K-05) with the Luminex 200 multiplex assay system built on xMAP technology (Millipore, USA). The results were calculated as pg per mg protein, and the bars were represented as units of IL-6 adjusted to control, taken as %.

### Glucose uptake assays

Glucose uptake was determined using a colorimetric assay kit (MAK083, Sigma-Aldrich, St. Louis, MO, USA). Briefly, ADSCs were differentiated in 12-well plates as described in the “Cell culture and incubation” section. Differentiated adipocytes were washed twice with PBS and then starved overnight in serum-free medium. Then, the cells were washed 3 times with PBS and glucose starved by incubating for 40 min in KRPH buffer (5 mM Na_2_HPO_4_, 20 mM HEPES, pH 7.4, 1 mM MgSO_4_, 1 mM CaCl_2_, 137 mM NaCl and 4.7 mM KCl) containing 2% BSA. Glucose uptake was assessed in the presence or absence of 10 mM 3-AT for 24 h with 1 mM 2-deoxy-D-glucose in KRPH for 20 min at 37°C and 5% CO_2_. As a positive control, the cells were stimulated with insulin (1 μM) for 20 min in the presence or absence of 3-AT. Glucose uptake levels were expressed in pmol/well.

### Protein kinase A (PKA) activity assay

Human differentiated adipocytes were incubated in the presence or absence of 10 mM 3-AT for 24 h, and cell lysates were obtained with PLB as previously described. Then, Protein kinase A (PKA) activity was determined using the PKA activity kit (ADI-EKS-390A, Enzo Life Science, Switzerland) after incubating the cell lysates with or without cAMP (0.1 mM) for 15 min.

### Statistical analysis

All experiments were repeated at least three times. In each independent experiment, two replicates were performed. The data are expressed as the mean ± standard error of the mean (SEM). Significant differences in the levels of *CAT* expression during the adipogenic differentiation, catalase activity, intracellular H_2_O_2_, SOD activity, GPX activity, gene expression, protein expression, lipolysis and glucose uptake were determined using the non-parametric Mann-Whitney U test. Statistical significance was defined as *P<0.05 and **P<0.01. Statistical analyses were performed using SPSS version 22 for Windows (SPSS, Chicago, IL, USA).

## Results

### CAT expression during adipogenic differentiation

First, we tested *CAT* expression in human ADSCs and differentiated adipocytes by analyzing gene and protein expression on different days during adipogenic differentiation. As expected, we found that *CAT* gene expression and protein levels were significantly up-regulated on days 5, 9 and 12 compared with day 0 (Figs A and B in S2 File, respectively). Moreover, CAT activity was significantly increased during adipogenic differentiation on days 5, 9 and 12 compared with day 0 (Fig C in S2 File).

### Inhibition of catalase activity by 3-amino-1,2,4-triazole (3-AT)

**Fig 1:**
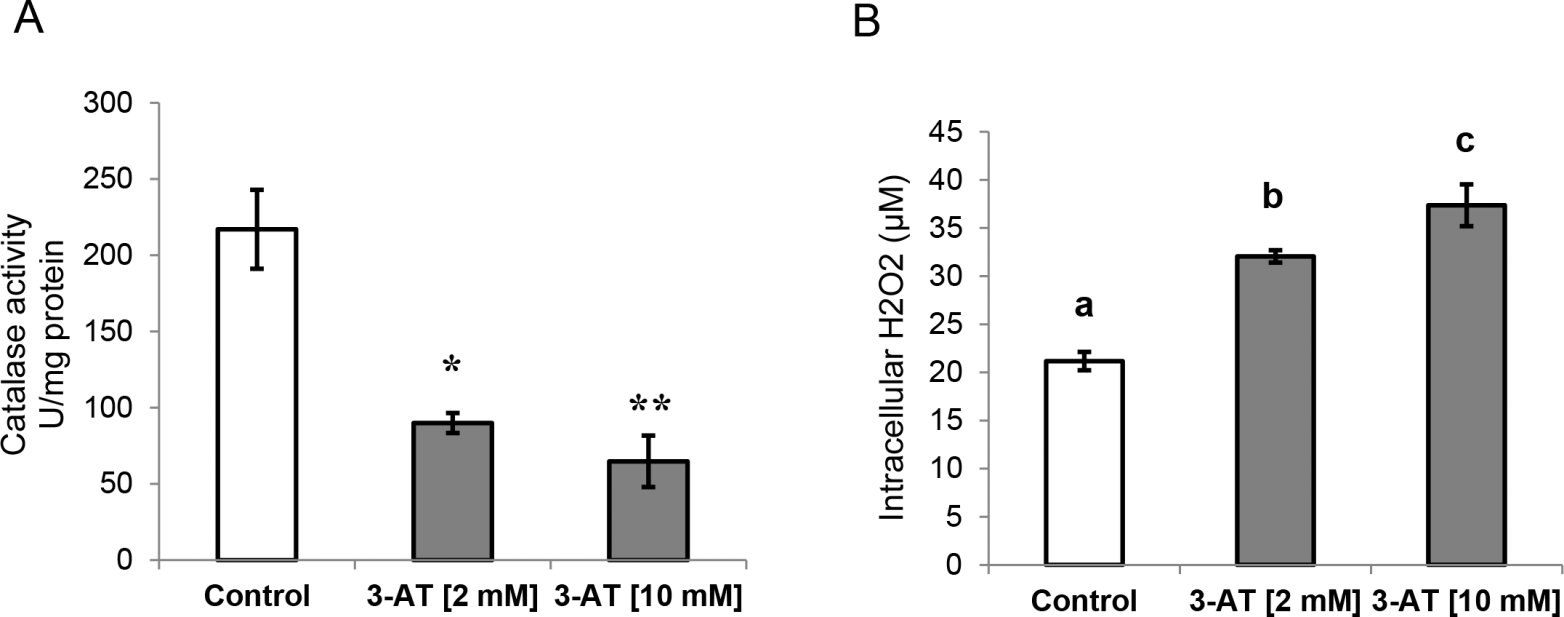
**Catalase activity (U/mg protein) (A) and intracellular H_2_O_2_ levels (μM) (B) in differentiated adipocytes.** Differentiated adipocytes at day 10 were treated with 2 mM and 10 mM 3-amino-1,2,4-triazole (3-AT) for 24 h. Significant differences were identified by Mann-Whitney U test. The data are expressed as the mean ± SEM. * *P*< 0.05 vs. control; ** *P*< 0.01 vs. control. Different letters indicate significant differences (*P*<0.05).

To characterize the toxicity of 3-AT in human differentiated adipocytes, we monitored the cellular viability in adipocytes exposed to increasing concentrations of 3-AT (0, 2, 6, 10, 50 and 100 mM) for 24 h using a Neubauer chamber and trypan blue (4%). No toxicity was observed for the tested range of 3-AT (S3 File). Then, taking into consideration this information as well as the available literature [28, 29, 30], we chose the concentrations of 2 mM and 10 mM 3-AT to test their effects on catalase activity in human differentiated adipocytes. 3-AT significantly inhibited catalase activity in a dose-dependent manner, producing an analogous increase in H_2_O_2_ levels (Fig 1A and 1B, respectively). However, 10 mM 3-AT generated a significantly higher increase in H_2_O_2_ than 2 mM 3-AT. According to these results, the final chosen condition for the following experiments was 10 mM 3-AT for 24 h.

### Effects of 3-AT treatment on the intracellular antioxidant system in human adipocytes

**Fig 2:**
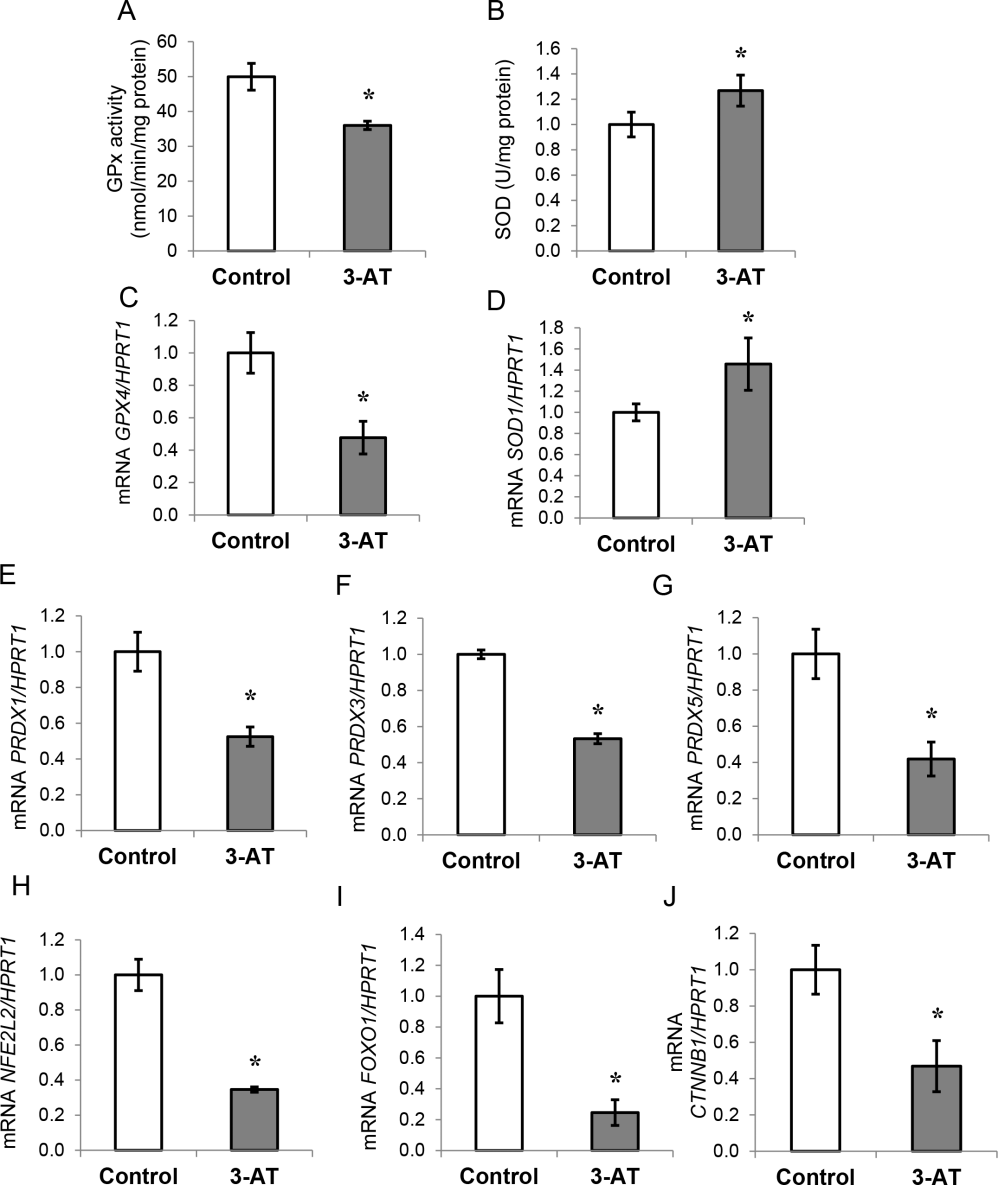
Effects of 3-amino-1,2,4-triazole (3-AT) on the antioxidant system in human differentiated adipocytes. A: Superoxide dismutase activity (SOD) expressed as U/mg protein in the presence or absence of 3-AT. B: mRNA expression of *SOD1* normalized to hypoxanthine-guanine phosphoribosyltransferase-1 (*HPRT1*) mRNA levels. C: Glutathione peroxidase (GPX) activity expressed as nmol/min/mg protein in the presence or absence of 3-AT. D: mRNA expression of glutathione peroxidase 4 (*GPX4*) normalized to *HPRT1* mRNA levels. E-G: mRNA expression of peroxiredoxin 1, 3 and 5 (*PRDX1*, *PRDX3*, and *PRDX5*) normalized to *HPRT1* mRNA levels in the presence or absence of 3-AT. H-J: mRNA expression of nuclear factor, erythroid 2-like 2 (*NRF2*), forkhead box O1 (*FOXO1*) and catenin beta 1 (*CTNNB1*) normalized to *HPRT1* mRNA levels in the presence or absence of 3-AT. The fold-changes from three independent experiments were calculated using the Pfaffl method. The data are presented as the means ± SEM of three independent experiments. Significant differences were identified using the Mann-Whitney U test; * *P*<0.05.

GPX activity, which is responsible for H_2_O_2_ degradation, decreased by 28% (Fig 2A; *P* = 0.001) in human adipocytes treated with 3-AT. Moreover, SOD activity, an enzyme that generates H_2_O_2_ from the degradation of superoxide anion, was significantly higher in 3-AT-treated adipocytes (Fig 2B). Similarly, *GPX4* gene expression was significantly inhibited, whereas *SOD1* expression was increased (*P*<0.05) after 3-AT treatment (Fig 2C and 2D, respectively). Interestingly, the gene expression levels of *PRDX1*, *PRDX3* and *PRDX5*, enzymes involved in H_2_O_2_ degradation, were all significantly reduced in 3-AT treated cells (Fig 2E, 2F and 2G, respectively). In addition, we observed a decrease in the gene expression levels of *NRF2*, *FOXO1* (*P*<0.05), (Fig 2E and 2F, respectively) and *CTNNB1* (Fig 2J).

### Effects of 3-AT treatment on inflammation in adipocytes

**Fig 3:**
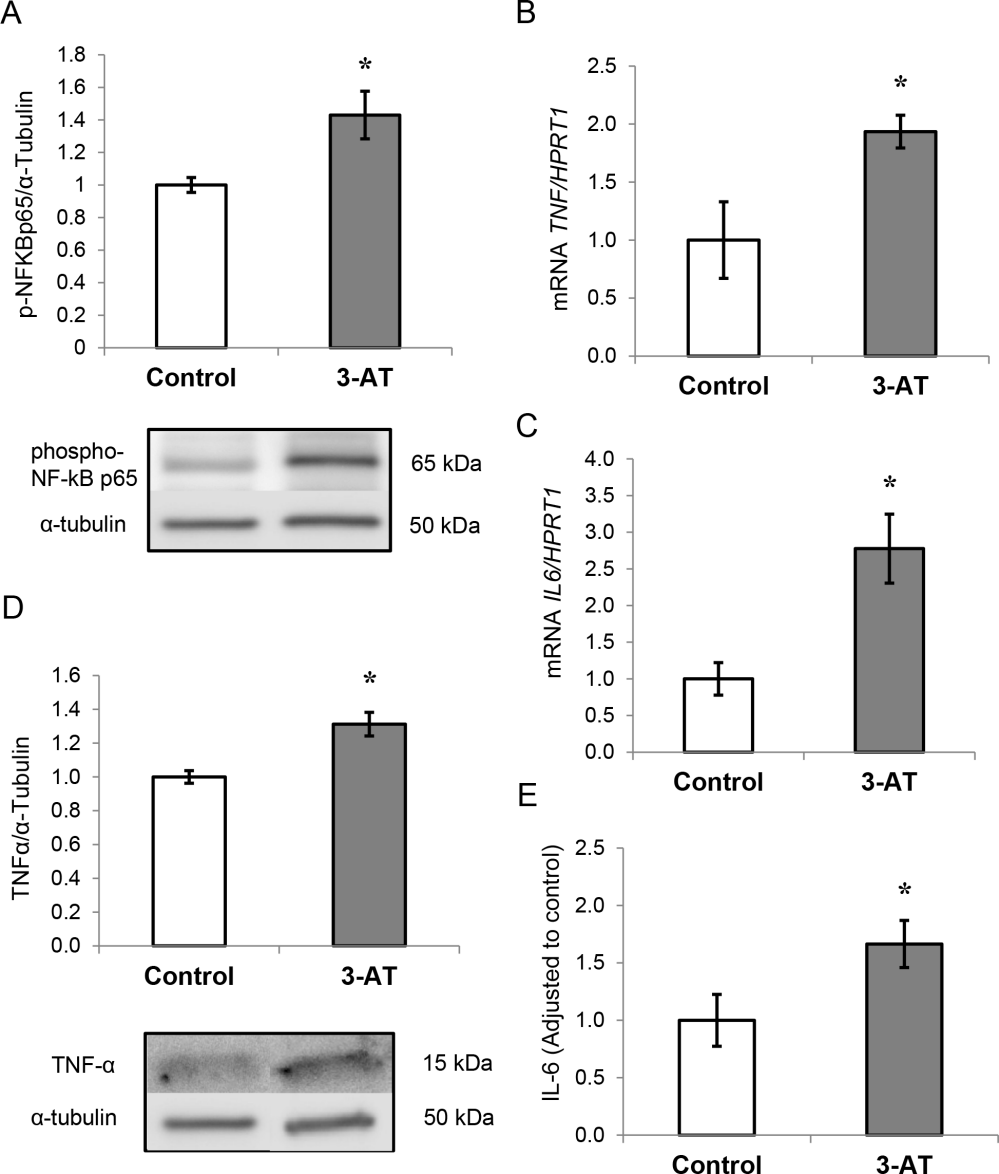
Effect of 3-amino-1,2,4-triazole (3-AT) treatment on inflammation in adipocytes. A: phospho-NFκB p65 protein levels analyzed by western blot. B: mRNA expression of tumor necrosis factor-α (*TNFA*) normalized to hypoxanthine-guanine phosphoribosyltransferase-1(*HPRT1*) mRNA levels. C: mRNA expression of interleukin 6 (*IL-6*) normalized to *HPRT1* mRNA levels. The fold-changes from three independent experiments were calculated using the Pfaffl method. D: TNF-α protein levels analyzed by western blot using a specific antibody, normalized to α-tubulin. E: IL-6 protein levels analyzed by XMap technology (Luminex) as indicated in the methods section. The data from three independent experiments are presented as the means ± SEM. Significant differences were identified using the Mann-Whitney U test; * *P<*0.05.

Next, we determined whether 3-AT-treated adipocytes displayed signs of inflammation by analyzing NF-κB activation and pro-inflammatory marker expression and synthesis. Interestingly, adipocytes treated with 3-AT had higher levels of the phosphorylated NF-κB p65 subunit (Fig 3A). In addition, the treatment of adipocytes with 3-AT significantly increased *TNF* and *IL-6* gene expression levels (FC (fold-change) = 1.93, *P* = 0.03; FC = 2.77, *P* = 0.037, respectively) (Fig 3B and 3C, respectively). Moreover, TNF-α and IL-6 protein levels were significantly increased upon 3-AT treatment in human adipocytes (Fig 3D and 3E, respectively). However, the mRNA levels of *NFKB2* and *TNFRSF1A* did not change after treatment with 3-AT (data not shown).

### Effects of 3-AT on adipocyte metabolism

**Fig 4:**
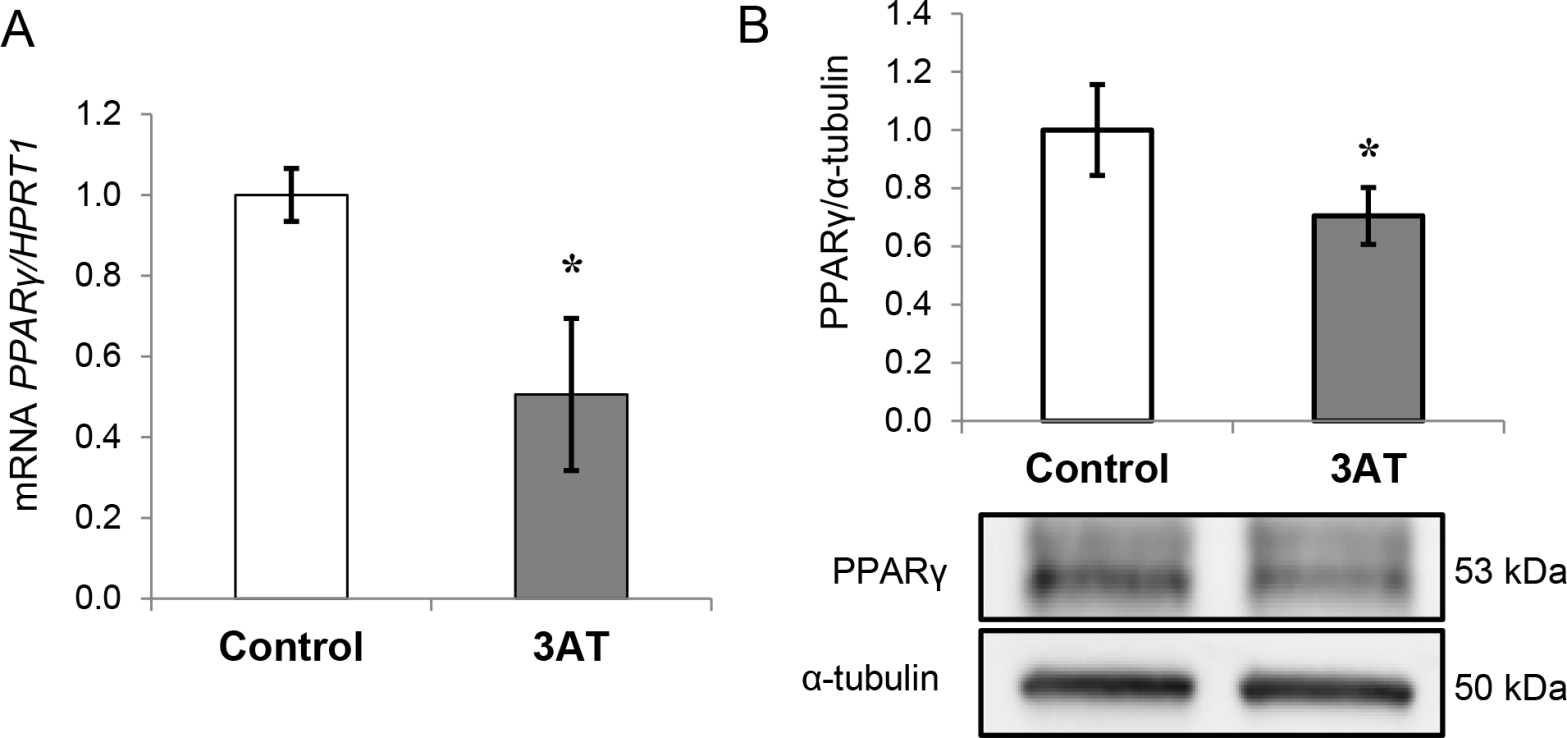
Effects of 3-amino-1,2,4-triazole (3-AT) on peroxisome proliferator-activated receptor gamma (PPARγ) expression. A: mRNA expression of *PPARγ* in human differentiated adipocytes. The mRNA levels were normalized to those of hypoxanthine-guanine phosphoribosyltransferase-1 (*HPRT1*) and the fold-changes from three independent experiments were calculated using the Pfaffl method. B: Protein expression of PPARγ analyzed by western blot as described in the Methods section. Protein levels were normalized to the internal control α-tubulin and expressed as fold-changes. The data are presented as the means ± SEM of three independent experiments. Significant differences were identified using the Mann-Whitney U test; * *P<*0.05, ** *P<*0.01.

To characterize the underlying regulation of these findings, we analyzed the gene and protein expression levels of *PPARγ*, which is involved in both lipid and glucose metabolism, and we found that they were down-regulated after 3-AT treatment (Fig 4A and 4B, respectively).

#### Glucose metabolism

**Fig 5:**
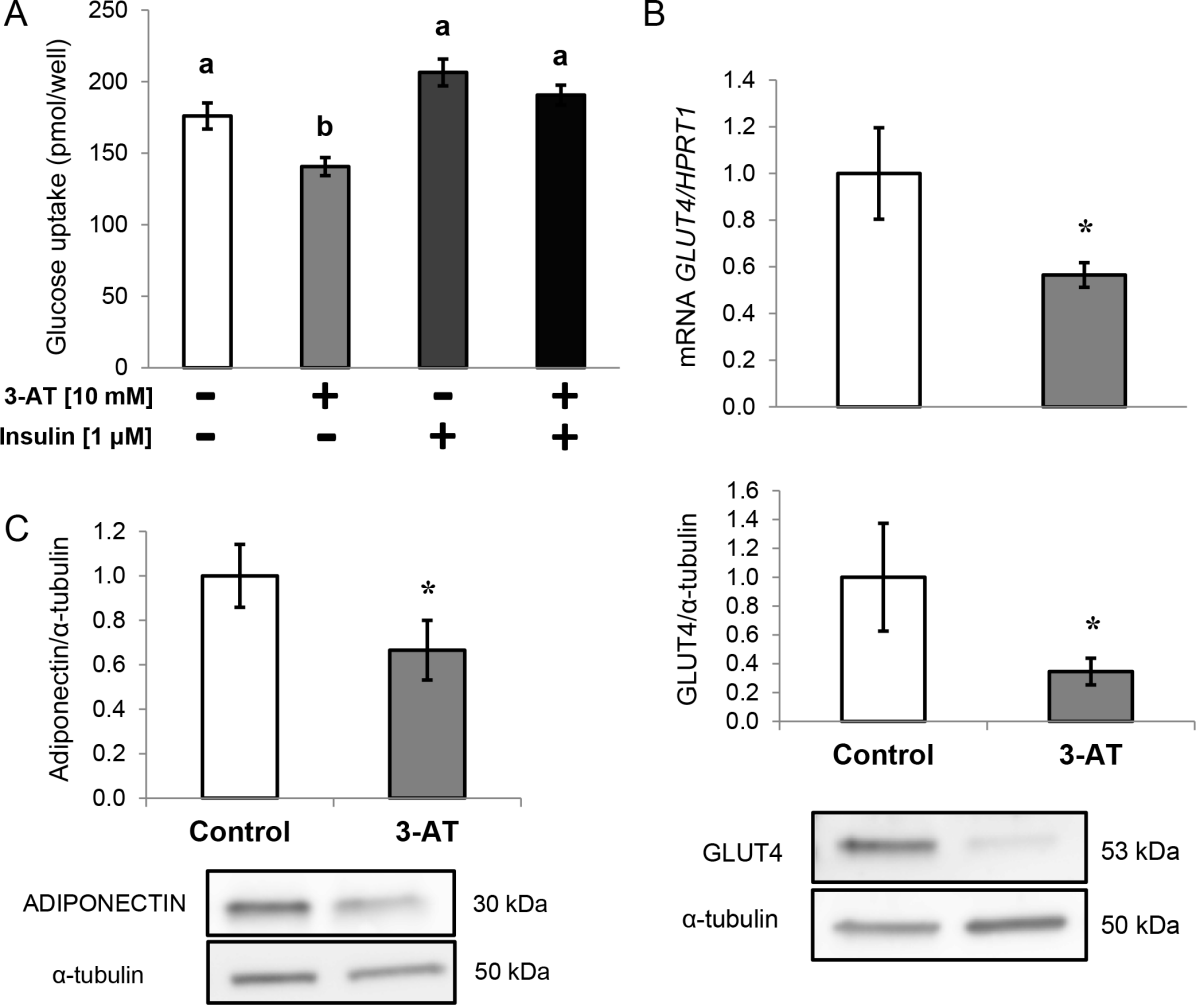
Effects of 3-amino-1,2,4-triazole (3-AT) on glucose metabolism in human differentiated adipocytes. A: Basal and insulin-stimulated glucose uptake levels in human differentiated adipocytes with or without 10 mM 3-AT and 1 μM insulin. Significant differences were identified using the Kruskal-Wallis test. B: mRNA and protein levels of glucose transporter 4 (*GLUT4)* in the presence or absence of 3-AT (10 mM, 24 h). The mRNA levels were normalized to those of hypoxanthine-guanine phosphoribosyltransferase-1 (*HPRT1*). The results are presented as fold-changes, which were calculated using the Pfaffl method. Protein expression of GLUT4 analyzed by western blot as described in the Methods section. Protein levels were normalized to the internal control (α-tubulin) and expressed as fold-changes. The data from three independent experiments are presented as the means ± SEM. Significant differences were identified using the Mann-Whitney U test. * *P*<0.05.

Basal glucose uptake was significantly inhibited by approximately 40% in human differentiated adipocytes after treatment with 3-AT (Fig 5A). However, insulin-stimulated glucose uptake did not change between conditions. This decrease in basal glucose uptake levels was accompanied by significantly lower *GLUT4* gene and protein expression after 3-AT treatment (Fig 5B). Moreover, the intracellular levels of adiponectin were also significantly lower in 3-AT-treated adipocytes compared with untreated cells (Fig 5C).

#### Lipid metabolism

**Fig 6:**
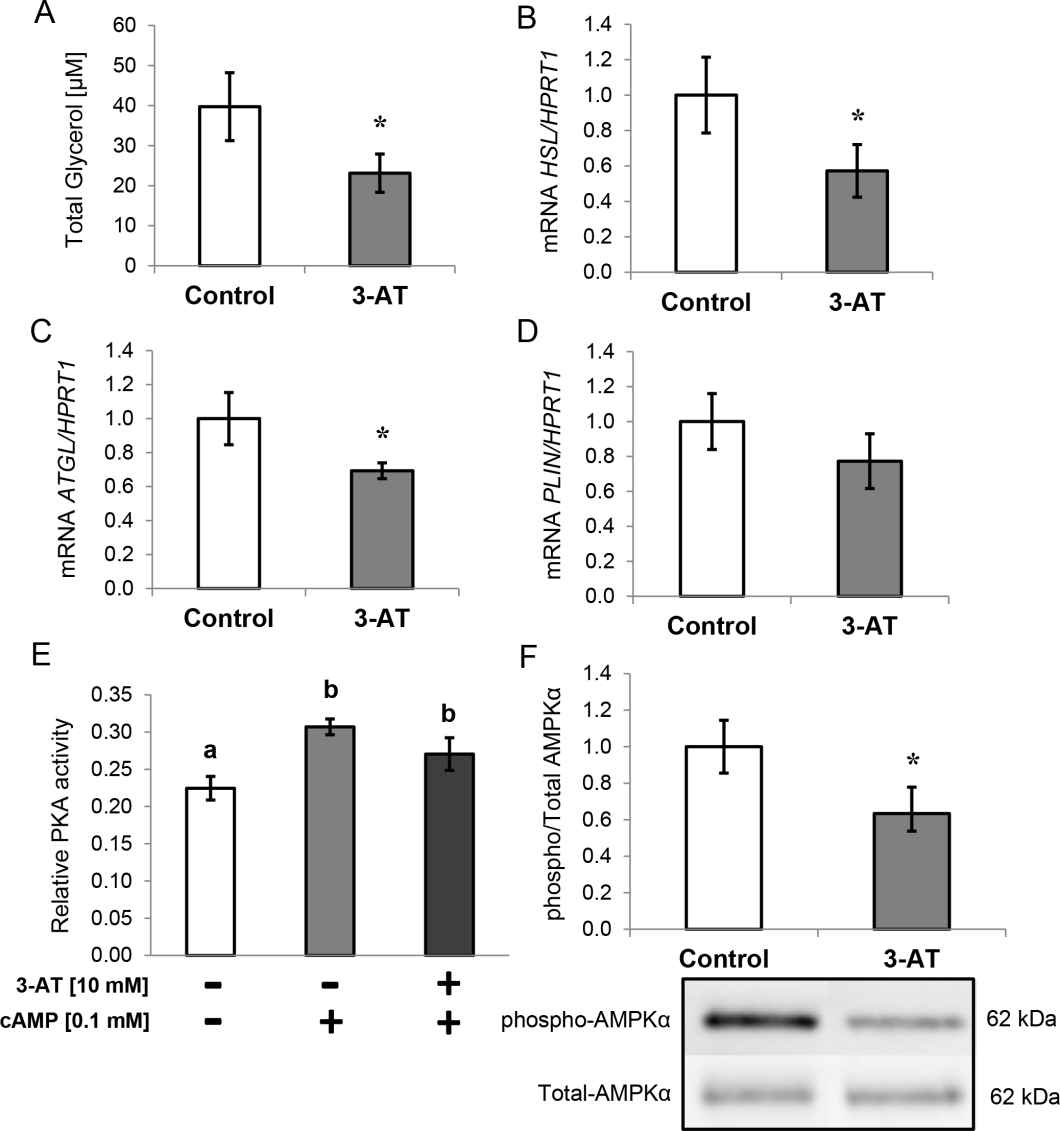
Effects of 3-amino-1,2,4-triazole (3-AT) on lipid metabolism. A: Glycerol levels (μM) in cell supernatants after treatment with 3-AT (10 mM, 24 h). B: Hormone sensitive lipase (*HSL*) gene expression. C: Adipose triglyceride lipase (*ATGL*) gene expression. D: Perilipin (*PLIN*) gene expression. The mRNA levels were normalized to those of hypoxanthine-guanine phosphoribosyltransferase-1 *(HPRT1*), and data from three independent experiments are presented as the means ± SEM of the fold-changes calculated using the Pfaffl method. E: Protein Kinase A (PKA) activity in the cell lysates of adipocytes treated with or without 3-AT (10 mM, 24 h) and 0.1 mM cAMP. F: 5'-AMP-activated protein kinase catalytic subunit alpha (AMPKα) protein levels. The cell lysates were prepared and then analyzed by western blot using specific antibodies against total AMPKα and phospho-AMPKα (Thr172) as described in the Methods section. The data are presented as the ratio of phosphor-AMPKα/total-AMPKα to no treatment fold-change, and the bars represent the means ± SEM of three separate experiments. Significant differences were identified using the Mann-Whitney U test; * *P<*0.05, ** *P<*0.01.

Regarding lipid metabolism, 3-AT-treated adipocytes exhibited reduced lipolysis as observed by decreased extracellular glycerol levels (Fig 6A). In addition, the expression levels of the lipases *HSL* and *ATGL* were significantly down-regulated upon 3-AT treatment (Fig 6B and 6C, respectively). The expression of *FABP4* was also significantly inhibited at both the mRNA and protein levels (31%↓, P = 0.037; 55%↓, P = 0.014, respectively). However, there were no significant differences in *PLIN* expression between 3-AT-treated and untreated adipocytes (Fig 6D).

Moreover, PKA activity was determined in the presence or absence of 3-AT (10 mM for 24 h) and cAMP (0.1 mM) in the differentiated adipocytes. Although 3-AT showed a tendency toward lower PKA activity when cAMP was added, the change was not statistically significant.

Finally, total AMPKα (catalytic subunit alpha) and phosphorylated AMPKα (Thr 172) were determined by western blot in the presence or absence of 3-AT. The ratio of phosphor-AMPKα to total AMPKα was significantly lower after treatment (Fig 6F).

## Discussion

**Fig 7:**
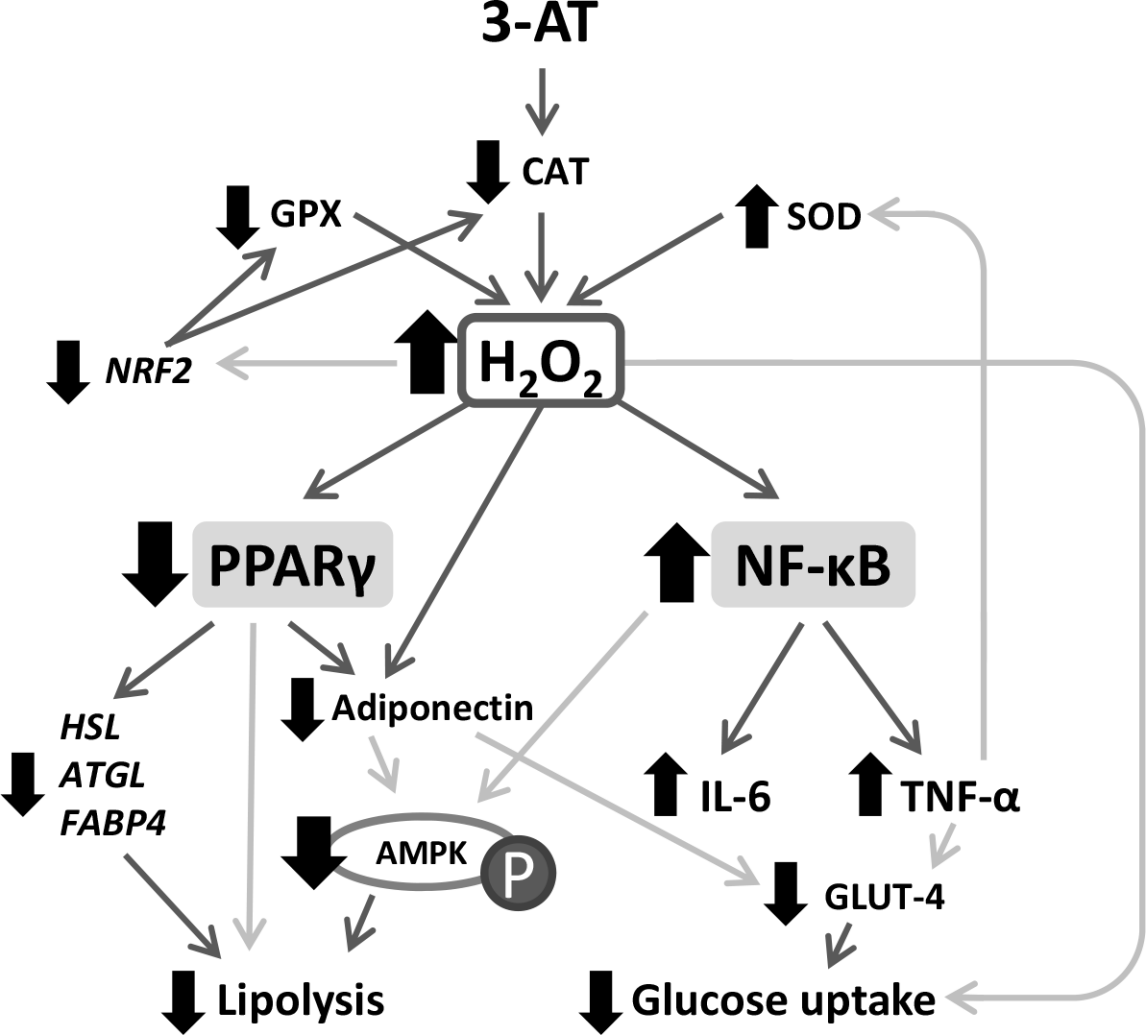
Diagram of the conditions observed in 3-AT-treated adipocytes. The increased hydrogen peroxide production leads to lower *PPARγ* expression and NF-κB activation, while decreasing the expression of important enzymes in metabolism, all of which finally reduce glucose uptake and lipolysis. AMPK: 5'-AMP-activated protein kinase; ATGL: adipose triglyceride lipase; CAT: catalase; FABP4: fatty-acid binding protein 4; GLUT4: glucose transporter type 4; GPX: glutathione peroxidase; HSL: hormone sensitive lipase; IL-6: interleukin 6; NRF2: nuclear factor, erythroid 2-like 2; NF-κB: nuclear factor of kappa light polypeptide gene enhancer in B-cells 2; P: enzyme phosphorylation; PPARγ: peroxisome proliferator-activated receptor gamma; SOD: superoxide dismutase; TNF-α: tumor necrosis factor alpha. The black arrows indicate an increase or decrease in activity, expression or protein levels. The bold arrows indicate causal relationships between findings, and the light grey arrows indicate potential relationships according to the revised literature.

Aiming to replicate the conditions observed in obesity, where CAT is inhibited, we intended to elucidate the degree to which catalase activity is responsible for the alterations found in obese adipose tissue using the inhibitor 3-AT in human adipocytes. The present study shows that the 10 mM 3-AT (24 h) treatment lowered CAT activity to 30% of the activity of control cells, doubled the content of cellular H_2_O_2_ and triggered inflammation while affecting antioxidant enzyme expression and lipid and glucose metabolism (Fig 7).

### 3-AT-treated adipocytes present an impaired antioxidant defense system

#### Activity and expression of antioxidant enzymes

The mechanisms involved in H_2_O_2_ synthesis and degradation were affected in 3-AT-treated adipocytes. We observed higher SOD activity, responsible for the conversion of superoxide ions into H_2_O_2_, and significantly lower GPX activity, and both events were paralleled by analogous changes in *SOD1* and *GPX4* gene expression. These results are in line with those of Than *et al*. [31] who found lower *GPX* and *CAT* protein expression levels in the presence of TNF-α-induced ROS in human adipocytes. The divergent results regarding SOD and GPX activities may seem counterintuitive. However, SOD appears to be the first line of defense against ROS in cells because it has been found augmented in different situations as a mechanism of protection [32,33,34], even along with lower CAT activity [35]. Other studies have also observed similar results where SOD activity was increased, while CAT [36,37] and GPX [37] activities were decreased. Moreover, *SOD* expression has been observed to be increased by TNF-α in a mechanism involving NF-κB activation [38]. Although we cannot rule out the possibility of partial inhibition of GPX by 3-AT [39], most of the studies using 3-AT report a specific inhibition of catalase [40,41]. Additionally, the expression levels of *PRDX1*, *PRDX3* and *PRDX5*, which are enzymes that are also involved in H_2_O_2_ clearance, were significantly reduced after treatment with 3-AT, which could contribute to increase H_2_O_2_ levels. Indeed, *PRDX3* is expressed in mature adipocytes and has been observed to be decreased in obesity [42,43]. In fact, Huh *et al*., 2012 demonstrated that *PRDX3* deficiency leads to impaired glucose metabolism and decreased adiponectin levels.

#### Transcription factors involved in the protection against oxidative stress

To elucidate the cause of the general decrease in antioxidant enzyme gene expression, we studied Nrf2 and FOXO1, two transcription factors responsible for the maintenance of redox homeostasis [44,24], as well as β-catenin, which is involved in oxidative stress responses [45]. Interestingly, we found that their expression levels were inhibited in 3-AT-treated adipocytes. First, our results agree with a recent study that found that H_2_O_2_ lowers *NRF2* expression [46]. Moreover, the inflammatory response present in adipocytes treated with 3-AT could also explain the reduced *NRF2* expression because it has been widely demonstrated that Nrf2 and NF-κB behave antagonistically [47]. Indeed, a similar situation is observed in chronic kidney disease in which Nrf2 is inactive, while NF-κB triggers inflammation [48]. Additionally, the lack of Nrf2 in obese mice resulted in severe metabolic syndrome [49]. The reduced *NRF2* expression could explain the lower mRNA levels of the antioxidant enzymes *GPX4*, *CAT*, and *PRDXs*, which are under transcriptional control by Nrf2 [44,23]. Regarding *SOD1*, its transcription is also regulated by other factors, which include TNF-α [38], as mentioned above, and could be increased as a mechanism of protection against 3-AT-induced oxidative stress. Second, the lower FOXO1 mRNA levels could also explain the inhibition of the gene expression of antioxidant enzymes that is observed in 3-AT-treated adipocytes [24]. Moreover, the gene expression of β-Catenin (*CTNNB1*), necessary for FOXO1 transcriptional activation [50], was inhibited by 3-AT treatment. A similar situation was observed in a previous study, which showed that ROS inhibits the Wnt/β-Catenin pathway and that this effect can be repaired by *CTNNB1* overexpression [45]. Interestingly, *CTNNB1* expression is also inhibited in mesenchymal stem cells from infants born to obese mothers [51].

### 3-AT treatment triggers inflammation and lowers *PPARγ* expression in adipocytes

The oxidative stress derived from 3-AT treatment led to two main events that could be the cause of the effects found in the present study. On one hand, 3-AT treatment triggered inflammation. On the other hand, the generated oxidative stress affected *PPARγ* expression, a transcription factor with important regulatory functions in oxidative stress and adipocyte metabolism

Regarding inflammation, the oxidative stress caused by 3-AT led to the activation of NF-κB and to the subsequent increase in transcription and protein synthesis of *TNF-α* and *IL-6*, which is in agreement with previous studies [15]. Indeed, it has been shown that there is a relationship between TNF-α, oxidative stress and insulin resistance [52].

Regarding PPARγ, we found its expression and protein levels to be significantly reduced after 3-AT treatment. In line with this finding, a previous study showed that H_2_O_2_ treatment significantly decreased the mRNA abundance of *PPARγ* in adipocytes, even suggesting that PPARγ could mediate the actions of H_2_O_2_ [19]. Similarly, ROS exposure led to lower *PPARγ* expression in murine adipocytes [4], and treatment with PPARγ agonists increased CAT protein levels in adipose tissues of obese individuals [21] and in 3T3-L1 cells [20].

Both inflammation and inhibition of *PPARγ* expression derived from the 3-AT treatment had a clear impact on the adipocyte’s metabolism, approaching to the situation found in obese adipose tissue.

### Effects of 3-AT treatment on glucose metabolism

We observed a significantly lower basal glucose uptake in 3-AT-treated adipocytes, which was accompanied by lower *GLUT4* gene and protein expression. However, the insulin-stimulated glucose uptake results indicate that insulin resistance is not present in the studied conditions. Nevertheless, our results are in line with those of Rudich *et al*., who observed that GLUT4 translocation was inhibited by ROS in 3T3-L1 murine adipocytes [6]. Furthermore, it has been shown that impaired PPARγ action is associated with insulin resistance [53]. Additionally, adiponectin, the insulin-sensitizing adipokine found at low circulating levels in obesity [54] that has been observed to increase *GLUT4* expression [55,56], was found at lower levels in 3-AT treated cells. Because adiponectin expression is enhanced by PPARγ agonists [57], the decreased *PPARγ* gene expression and the increased H_2_O_2_ concentrations could partially explain the lower adiponectin and glucose uptake levels observed in the 3-AT-treated cells. Indeed, similar results from other studies have shown that oxidative stress decreases the secretion of adiponectin [6,31,58,59]. However, another cause for the reduced glucose uptake could be TNF-α because it has been observed to cause a reduction in *GLUT4* expression in 3T3-L1 adipocytes [60]. In fact, artificially induced oxidative stress increased TNF-α production and provoked insulin resistance through reduced *GLUT4* expression in adipocytes [17]. Moreover, CAT prevented TNF-α-induced insulin resistance in 3T3-L1 adipocytes [61].

#### Effects of 3-AT treatment on lipid metabolism

We observed reduced lipolysis in 3-AT-treated adipocytes. To fully characterize the agents involved in regulation of lipolysis, PKA activity, AMPK activation and expression of lipase genes were determined. It has been shown that H_2_O_2_ oxidizes PKA cysteine residues, interfering with cAMP activation of the enzyme and thus reducing lipolysis [62,14]. However, we did not find differences in cAMP-stimulated PKA activity between control and 3-AT-treated adipocytes. In contrast, the reduced AMPK activation is in agreement with our lipolysis results. In fact, the decreased AMPK activation could actually be due to the existing inflammation [63] and to the lower adiponectin and lipolysis levels, which lead to AMPK phosphorylation [64,22]. In addition, the reduced lipolysis could also be derived from the lower gene expression levels of lipases *HSL* and *ATGL*. This could be explained by the lower *PPARγ* expression because HSL [65] and ATGL [66] are two of its transcriptional targets. Indeed, in a similar study, *PPARG2* knockout adipocytes were shown to exhibit reduced lipolysis [67]. Finally, although our results contradict with previous studies in which TNF-α has been shown to activate lipolysis [18], this could be due to inflammation being at early stages because we did not observe changes in *NFKB2* and *TNFRSF1A* expression in 3-AT-treated adipocytes [68].

## Conclusions

The present study shows that CAT activity is important for the metabolic homeostasis of adipocytes. In adipocytes, 3-AT treatment inhibited catalase activity and generated oxidative stress and inflammation while decreasing *PPARγ* expression, which led to lower glucose uptake and lipolysis. Our findings suggest an important role of catalase in the protection of adipocytes against the oxidative stress and metabolic complications observed in obesity through the regulation of H_2_O_2_ levels, which exerts complex signaling functions.

Finally, we have shown that the compound 3-AT can be successfully used to inhibit CAT activity in adipocytes *in vitro* as a tool for the study of ROS in metabolic alterations associated with obesity. Further research is needed to better understand the metabolic effects of catalase in adipose tissue in the presence of obesity *in vivo*.

## Limitations

We are aware of several limitations of the present study. In particular, the use of differentiated adipocytes from human ADSCs is not the same as the use of adipose tissues or mature adipocytes. Moreover, the degree to which we inhibited CAT (by approximately 70%) is higher than the inhibition of CAT activity observed in obese individuals (approximately 42%) [11]. Finally, the effects of 3-AT on the actions of insulin or other hormones upon other metabolic parameters and cellular responses was not fully elucidated in the present study, and thus, requires further research.

## Supporting Information

S1 File Oil Red O staining of human adipose-derived stem cells at day 0 (d0) and during the adipogenic differentiation at days 5, 9 and 12 (d5, d9 and d12). Fig A: Optical microscopy images. Fig B: Quantification of lipid content from Oil Red O staining (absorbance at 520 nm). All values are expressed as the means ± SEM of three independent experiments. Significant differences were identified using the non-parametric Mann-Whitney U test; *P< 0.05; * *P*<0.01.
(PDF)

S2 File Catalase expression during the adipogeneic differentiation. Fig A: Catalase (*CAT*) mRNA levels normalized to those of glyceraldehyde 3-phosphate dehydrogenase (*GAPDH*) and presented as fold-change, calculated using the Pfaffl method. Fig B: CAT protein levels from cell lysates analyzed by western blot using a specific antibody against CAT, normalized to the internal control (α-tubulin) and expressed as fold-change. Fig C: CAT activity of cell lysates during adipogenic differentiation. All values are expressed as the means ± SEM of three independent experiments. Significant differences were identified using the non-parametric Mann-Whitney U test; * *P*< 0.05.
(PDF)

S3 File Effects of increasing concentrations of 3-amino-1,2,4-triazole (3-AT) on cell viability after a 24-h incubation. Cell viability was determined using a Neubauer chamber and trypan blue (4%).
(PDF)
