# Supplementary figures and images for: Impact of 3-Amino-1,2,4-Triazole (3-AT)-Derived Increase in Hydrogen Peroxide Levels on Inflammation and Metabolism in Human Differentiated Adipocytes
Source: PLoS One. 2016 Mar 29;11(3):e0152550. doi: 10.1371/journal.pone.0152550 (PMC4811533; doi:10.1371/journal.pone.0152550)

A

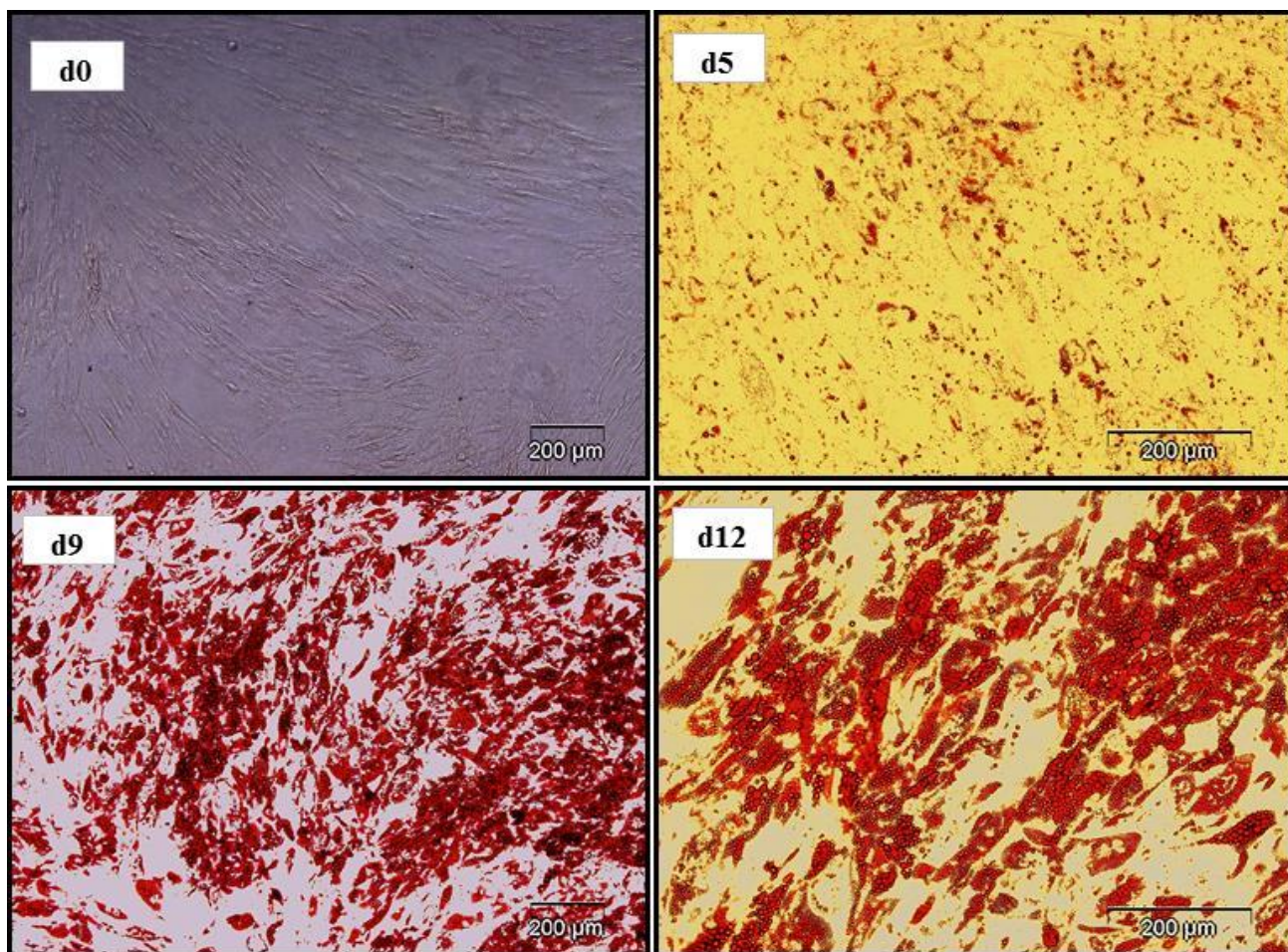

B

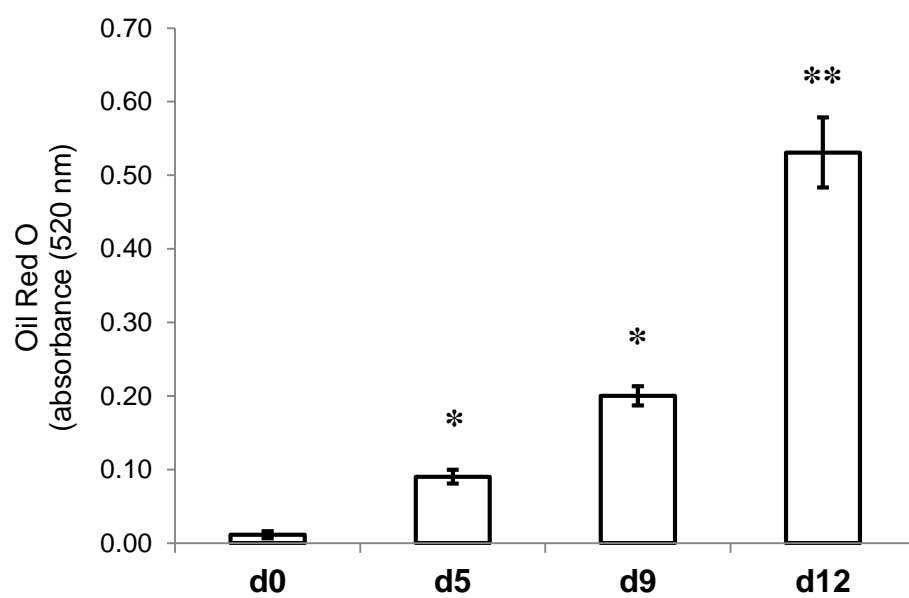

Supplement: S1 File — Fig A: Optical microscopy images. Fig B: Quantification of lipid content from Oil Red O staining (absorbance at 520 nm). All values are expressed as the means ± SEM of three independent experiments. Significant differences were identified using the non-parametric Mann-Whitney U test; *P< 0.05; * P<0.01. (PDF) [file pone.0152550.s001.pdf]

A

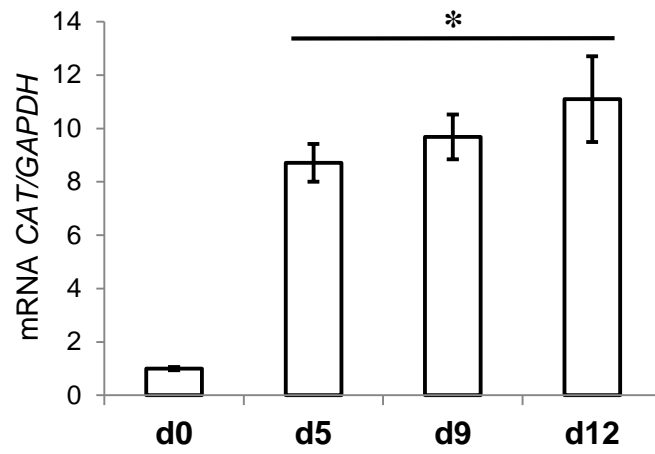

B

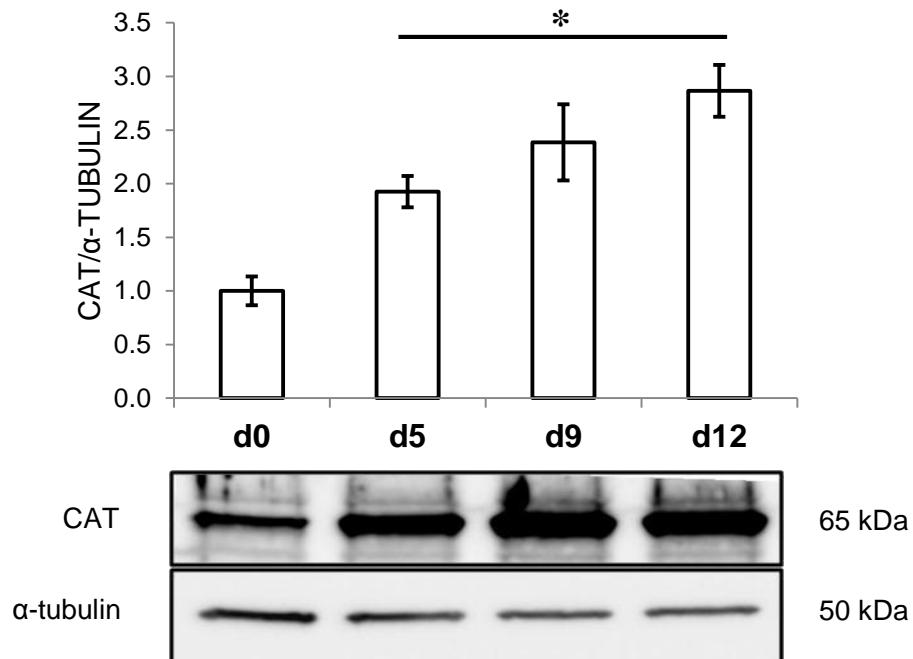

C

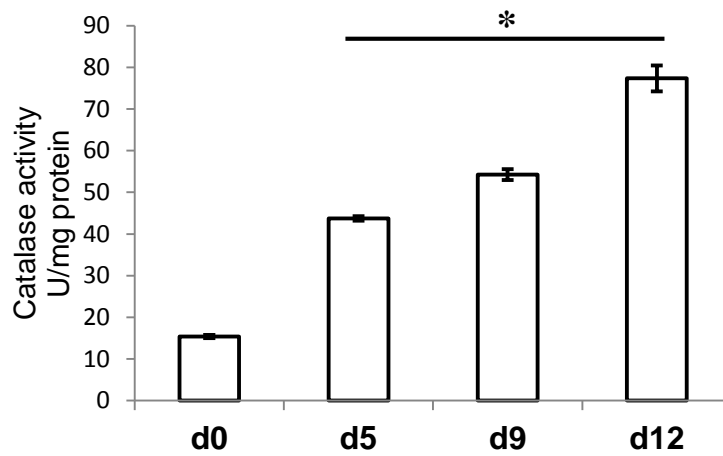

Supplement: S2 File — Fig A: Catalase (CAT) mRNA levels normalized to those of glyceraldehyde 3-phosphate dehydrogenase (GAPDH) and presented as fold-change, calculated using the Pfaffl method. Fig B: CAT protein levels from cell lysates analyzed by western blot using a specific antibody against CAT, normalized to the internal control (α-tubulin) and expressed as fold-change. Fig C: CAT activity of cell lysates during adipogenic differentiation. All values are expressed as the means ± SEM of three independent experiments. Significant differences were identified using the non-parametric Mann-Whitney U test; * P< 0.05. (PDF) [file pone.0152550.s002.pdf]

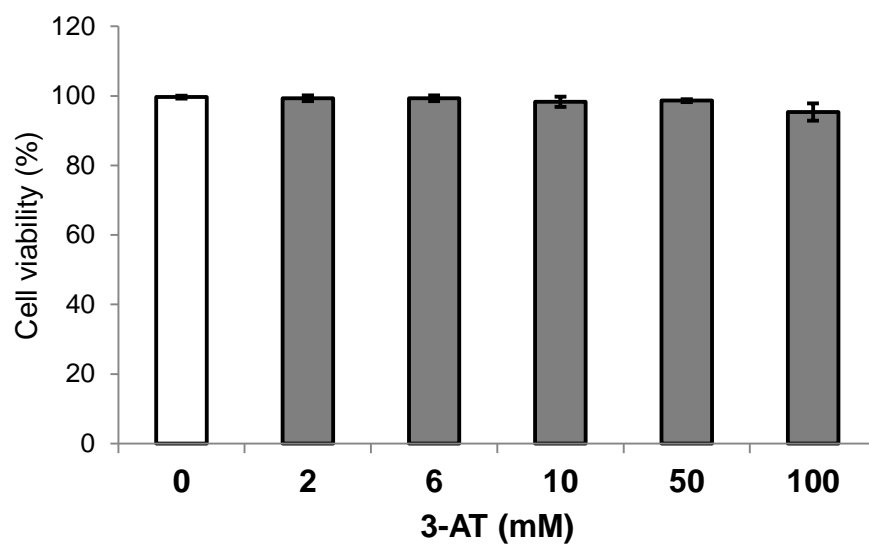

Supplement: S3 File — Cell viability was determined using a Neubauer chamber and trypan blue (4%). (PDF) [file pone.0152550.s003.pdf]
